# A Colloidal Singularity Reveals the Crucial Role of Colloidal Stability for Nanomaterials *In-Vitro* Toxicity Testing: nZVI-*Microalgae* Colloidal System as a Case Study

**DOI:** 10.1371/journal.pone.0109645

**Published:** 2014-10-23

**Authors:** Soledad Gonzalo, Veronica Llaneza, Gerardo Pulido-Reyes, Francisca Fernández-Piñas, Jean Claude Bonzongo, Francisco Leganes, Roberto Rosal, Eloy García-Calvo, Ismael Rodea-Palomares

**Affiliations:** 1 Departamento de Ingeniería Química, Universidad de Alcalá, Alcalá de Henares, Madrid, Spain; 2 Department of Environmental Engineering Sciences, University of Florida, Gainesville, Florida, United States of America; 3 Departamento de Biología, Facultad de Ciencias, Universidad Autónoma de Madrid, Madrid, Spain; 4 Instituto Madrileño de Estudios Avanzados (IMDEA) Agua, Alcalá de Henares, Madrid, Spain; Dowling College, United States of America

## Abstract

Aggregation raises attention in Nanotoxicology due to its methodological implications. Aggregation is a physical symptom of a more general physicochemical condition of colloidal particles, namely, colloidal stability. Colloidal stability is a global indicator of the tendency of a system to reduce its net surface energy, which may be achieved by homo-aggregation or hetero-aggregation, including location at bio-interfaces. However, the role of colloidal stability as a driver of ENM bioactivity has received little consideration thus far. In the present work, which focuses on the toxicity of nanoscaled Fe° nanoparticles (nZVI) towards a model microalga, we demonstrate that colloidal stability is a fundamental driver of ENM bioactivity, comprehensively accounting for otherwise inexplicable differential biological effects. The present work throws light on basic aspects of Nanotoxicology, and reveals a key factor which may reconcile contradictory results on the influence of aggregation in bioactivity of ENMs.

## Introduction

Environmental health and safety (EHS) is one of the key challenges in the field of nanotechnology [1]–[3]. Despite research efforts, EHS development is hampered by a series of difficulties, from misconceptions in the field, to the lack of agreement on methodological aspects of EHS execution [1], [4], [5]. The latter is primarily due to the peculiar and complex intrinsic characteristics of nano-sized materials [1], [3], [4], [6], [7]. Overall, more than a dozen physicochemical properties could potentially contribute to hazardous interactions at the bio-nano interface [3], [8], [9]. However, it is hard to find comprehensive studies where key drivers of ENM bioactivity are clearly identified [1]. Currently, the influence of aggregation on ENM bioactivity is surrounded by controversy, mainly due to its broad methodological implications and the existence of contradictory results [1], [4], [10]. Despite the importance of the effects of aggregation in EHS [4], [5], no international consensus exists on how to handle ENM aggregation [1], [4], [5]. Notably, Schorus & Lison [1] stated in their recent commentary “Focusing the research effort” that “the influence of SNPs (silica nanoparticles) aggregation in the biological response remains unclear and it remains impossible to state whether or not it is necessary to have a dispersion of SNPs before testing”. Although they revised SNPs toxicity data, their statement may be generalized to ENMs. Aggregation is a symptom of a more general physicochemical condition of colloidal particles, *i.e.*, colloidal stability [11]. Colloidal stability has been considered a major issue in EHS assessment of ENMs to warrant exposure and dosimetry [2], [4]. However, despite the growing body of evidence regarding biophysically mediated bioactivity of ENMs in a variety of model biological systems [8], [12]–[14], the role of colloidal stability as its driver has been essentially overlooked. In the present work, the spontaneous occurrence of an nZVI speciation phenomenon [3], [15], which we have termed *colloidal singularity*, showed that taking only colloidal stability into account allowed us to reconcile otherwise inexplicable biological effects.

## Results

### Physicochemical characterization of nZVI

**Figure 1:**
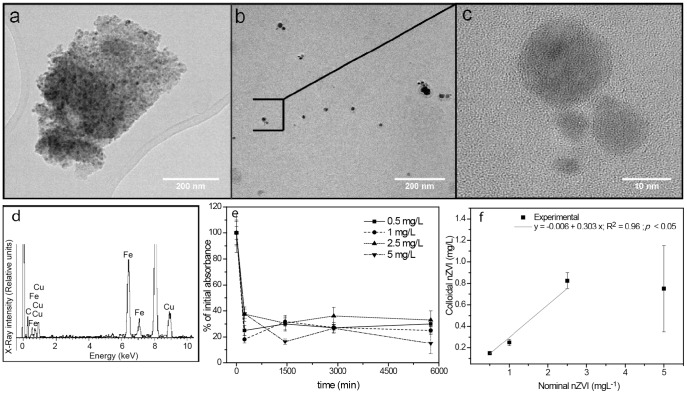
Characterization of nZVI working suspensions under experimental conditions. Representative TEM images of (a) nZVI aggregates in 50 mgL^−1^ nZVI stock suspensions, and (b) small nZVI aggregates and primary sized particles in diluted (5 mg/L) nZVI suspensions. (c) Detail of a small nZVI aggregate integrated by 4 agglomerated individual spherical particles. (d) XEDS spectra of (c), Fe is the main constituent. Ni and Cu intense signals in the XEDS spectra (when present), are artifacts due to the metallic grid supporting the samples, and of the sample holder of the TEM instrument, respectively. (e) Reduction of residual absorbance (λ = 750 nm) of nZVI suspensions as a result of aggregation and sedimentation at relevant experimental times (0 h, 4 h, 24 h, 48 h and 72 h). (f) Correlation between nominal nZVI concentration (mg/L) and nZVI concentration as relevant colloidal fraction (mg/L) in working suspensions at 72 h.

The surface area and mean particle size of pristine nZVI powder was 43.9 m^2^ g^−1^ and 20 nm, respectively, as calculated by the Nova 1200 BET (Brunauer –Emmett –Teller) method. Pristine nZVI powder showed a partial degree of surface oxidation as revealed by XRD analysis (**Table S1**). This is due to the high tendency of zero valent iron to oxidize in aerobic conditions [16], [17]. nZVI suspensions ranging from 2.5 to 50 mgL^−1^ were prepared in Milli-Q water and OECD TG 201 algal growth medium [18]. Their main physicochemical properties (**Table 1**) were studied to select the best option for working as a stock suspension for subsequent EHS characterization. General trends were as follows: nZVI appeared aggregated with effective diameters of approximately 100 nm in Milli-Q water, and between 200–400 nm in OECD medium. The Polydispersity index was below 0.5 in Milli-Q water, and ranged from 0.5 to 0.8 in OECD TG 201 medium, indicating a high degree of polydispersity in particle size, especially in OECD TG 201 medium. nZVI appeared with a near neutral surface charge when dispersed in Milli-Q water, however it showed a negative surface charge in OECD TG 201 medium, ranging from −17.54 mV for 2.5 mgL^−1^ to −27.5 mV for 25 mgL^−1^. The observed tendency of nZVI to acquire negative charge in saline medium has been associated with the high affinity of ions to be adsorbed onto the oxy-hydroxide surface shell of nZVI formed under oxidant conditions [17]. Primary particle size and morphology was investigated by Transmission electron microscopy (TEM) in nZVI suspensions prepared in Milli-Q water (**Figure 1**). 50 mgL^−1^ nZVI suspensions showed a high degree of agglomeration (**Figure 1.a**), however, the visualization of diluted suspensions (5 mgL^−1^) allowed us to observe primary sized particles and small particle aggregates (**Figure 1.b, c**). The mean size, measured by TEM, of primary aggregates were 37.5±9.7 nm, and that of individual small and big nZVI primary particles was 4.0±1.4 and 12.7±1.7 nm, respectively (**Figure S1**). XEDS (X-Ray Energy Dispersive Spectroscopy) analysis confirmed Fe as the main constituent of the visualized particles (**Figure 1.d**). 25 mgL^−1^ of nZVI in Milli-Q water had the lowest polydispersity index (PDI) and effective diameter (**Table 1**). Therefore, it was selected as stock suspension.

**Table 1:** Main physicochemical parameters of nZVI suspensions prepared in Milli-Q water and OECD algal growth medium.

nZVI (mgL^−1^)	Dispersion Medium	Effective diameter (nm)^1^	Polydispersity Index (PDI)	ζ-potential (mV)	Total surface area (m^2^ g^−1^)^3^	pH	Conductivity(µScm^−2^)
2.5	H_2_O	124.5±21.0	0.471	−8.5±5.5	7.19	6	0.011
	OECD medium	284.2±47.0	0.609	−17.5±2.3	3.15	8.2	0.214
5	H_2_O	82.1±13.5	0.407	−3.4±1.3	10.91	6	0.007
	OECD medium	183.4±27.4	0.816	−23.6±2.4	4.88	8.2	0.202
25	H_2_O	108.9±16.4	0.406	−5.55±2.2	8.22	6	0.0032
	OECD medium	121.3±16.1	0.522	−27.50±2.7	7.38	8.2	0.207
50	H_2_O	123.0±12.5	0.451	−7.0±3.3	7.28	6	0.012
	OECD medium	369.1±56.3	0.539	−22.2±2.5	2.43	8.2	0.206

Measurements were conducted at 25°C. Effective diameter and ζ-potential in milli-Q water were performed in the presence of 10 mM KCl. Data are expressed as mean ± standard deviation (SD) when applicable.

1 : Effective diameter (hydrodynamic diameter measured by dynamic light scattering (DLS).

3 : Total surface area (SSA) is calculated according to equation 1.

### nZVI working suspensions show a *colloidal singularity* in a narrow dose range

**Figure 2:**
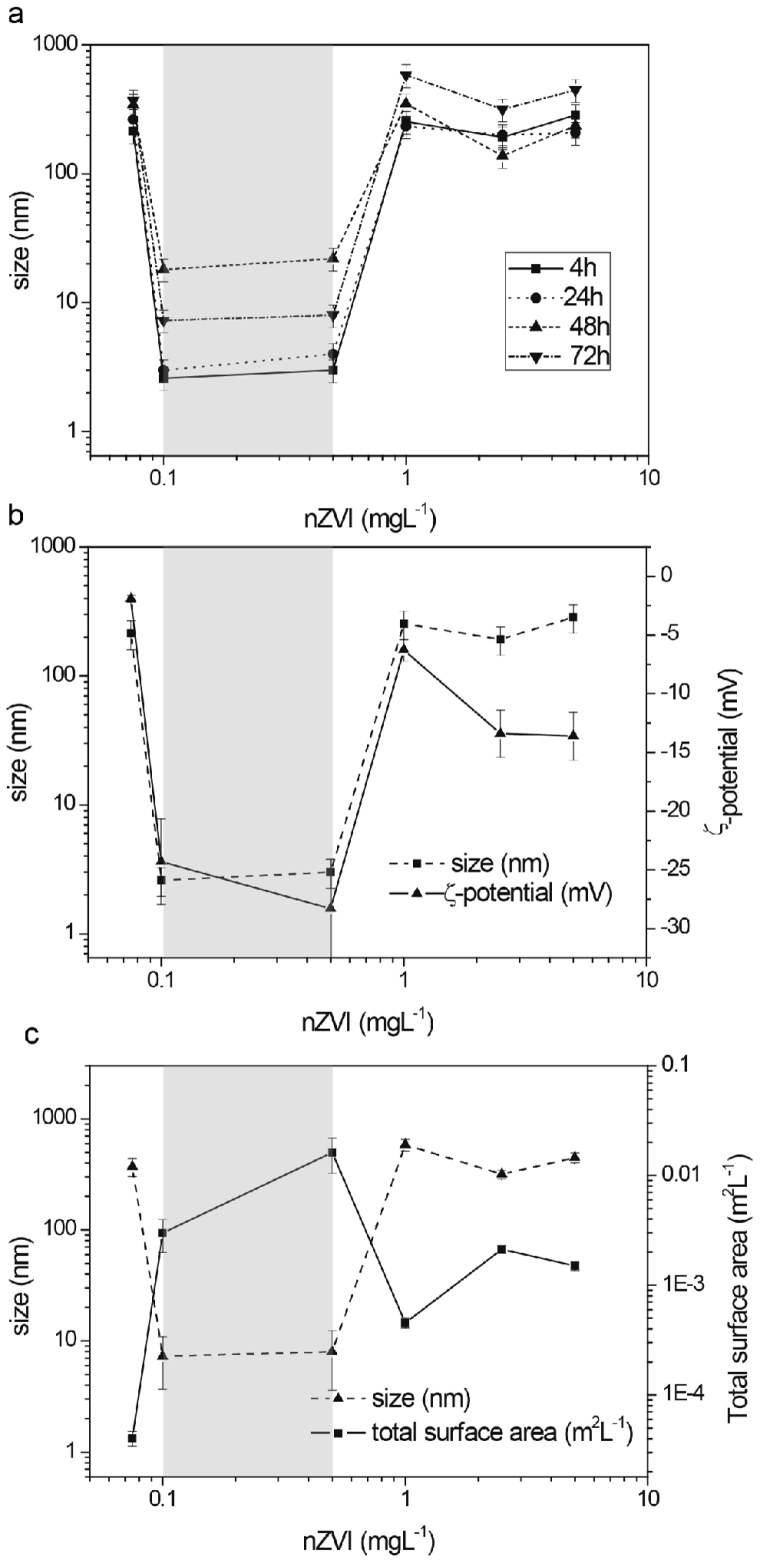
The occurrence of a *colloidal singularity*: Main physicochemical characteristics of colloidal nZVI fractions under experimental conditions. (a) Particle size of a range of nZVI concentrations at 4 h, 24 h, 48 h and 72 h. (b) ζ-potential and particle size for a representative exposure time (4 h). (c) Total surface area (m^2^ L^−1^) and particle size for a representative exposure time (4 h). The concentration range in which the *colloidal singularity* occurred is marked in blue.

The stability of the nZVI working suspensions used in EHS was evaluated under shaking conditions by measuring the residual absorbance (λ = 750 nm) at relevant experimental lapse times [4], [15] (0 h, 4 h, 24 h, 48 h and 72 h) (**Figure 1. e**). Extensive sedimentation of nZVI particles was observed within the first 4 h, in agreement with the high tendency of nZVI to homo-aggregate [19]–[21]. However, suspensions remained stable from 4 h to 72 h (**Figure 1.e**), confirming the relevant nZVI colloidal fractions in the exposure experiments [3], [15]. The actual nZVI concentrations as stable colloidal fractions in equilibrated conditions were 20–40% of nominal concentrations. They maintained a linear correlation (R^2^ = 0.95, *p*<0.05) with the initial dosage (**Figure 1. f**). Main physicochemical properties (e.g. size, surface charge and total surface area) of the stable colloidal nZVI fractions in the test system were monitored during 72 h (**Figure 2.a, b, c**). As a general trend, nZVI appeared in the form of aggregates of sizes around 150–500 nm, with ζ-potential around −15 mV and a total surface area of near 10^−3^ m^2^ L^−1^. However, nZVI presented a substantially different physicochemical state within a small dose range (0.1–0.5 mgL^−1^). In this narrow interval nZVI appeared suspended in its primary size (4–12 nm) along the experimental lapse time (**Figure 2.a**), with a substantially more negative surface charge (ζ-potential = −28 mV) (**Figure 2.b**), and a nearly 10 times higher total surface area (**Figure 2.c**). To our knowledge, such an extreme asymmetry in behavior of colloidal suspensions without the interplay of any external factor has never been previously reported. For simplicity, we will herein refer to this phenomenon as *colloidal singularity*.

### The *colloidal singularity* prevents nZVI toxicity

**Figure 3:**
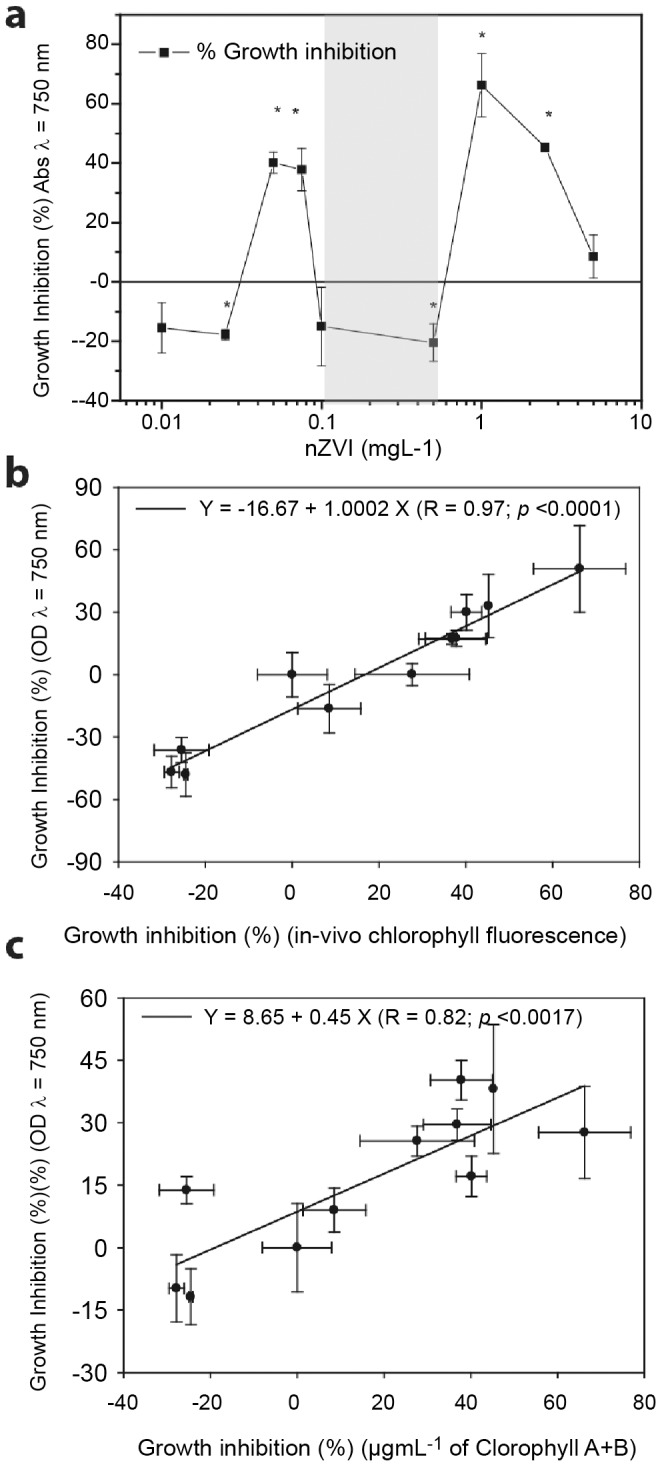
The *colloidal singularity* prevents nZVI toxicity. (a) Growth inhibition of *P. subcapitata* exposed to a linear nZVI dose gradient (0.025–5 mgL^−1^) based on OD_λ_ = _750 nm_ biomass surrogate. Typically, growth rate of control replicates was 1.4 d^−1^. The coefficient of variance for 72 h control cultures was 9%. Statistically significant differences (*p*<0.05) are marked by an asterisk. (b,c) Correlations of biomass surrogates for growth inhibition of *P. subcapitata* exposed to an nZVI dose gradient (0.025–5 mgL^−1^). The concentration range in which the *colloidal singularity* occurred is marked in blue.

**Figure 4:**
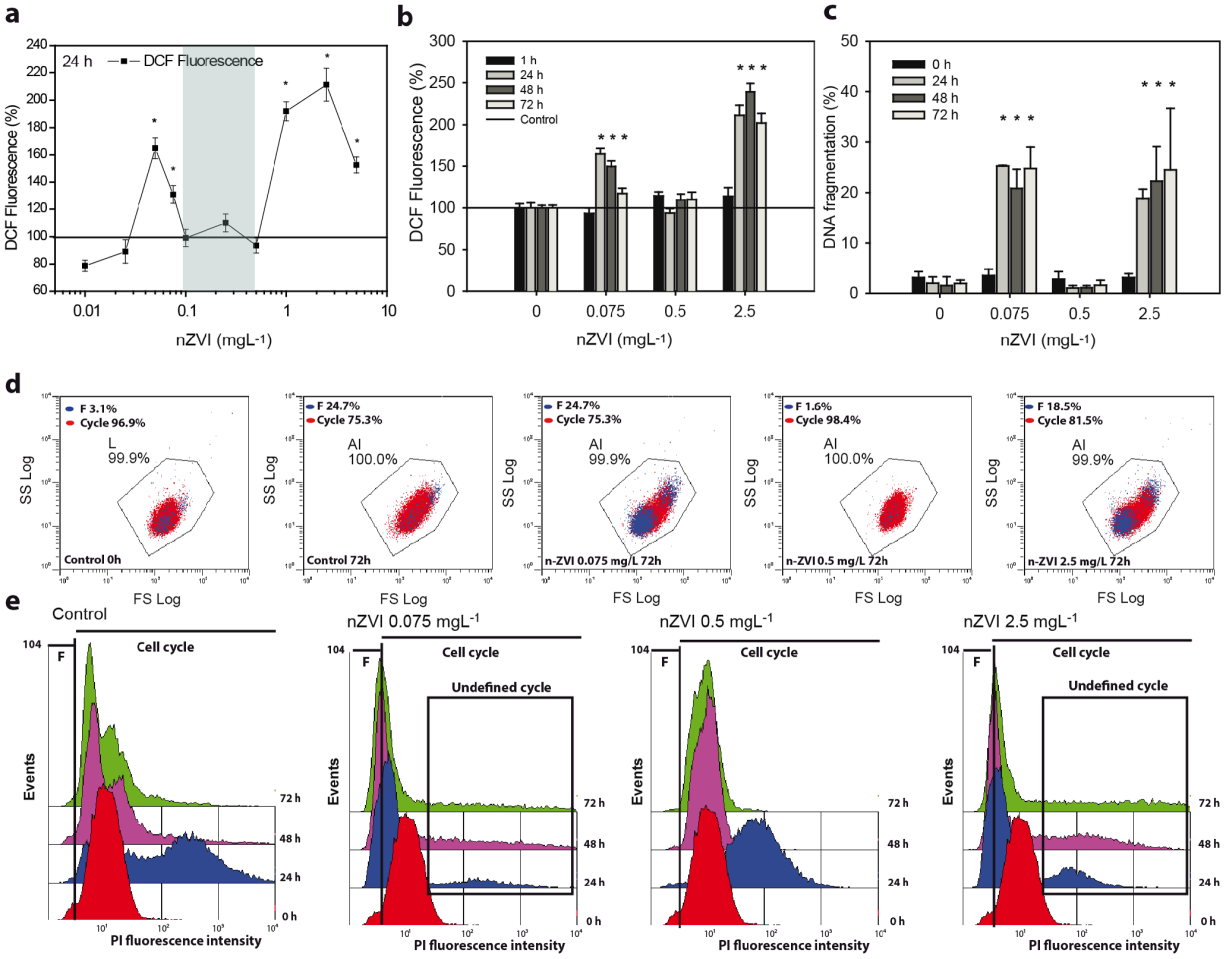
nZVI toxicity is consistently accompanied by ROS and cell cycle alterations. (a) Intracellular ROS formation as DCF fluorescence (% with respect to control levels) of *P. subcapitata* to an nZVI dose gradient (0.025–5 mgL^−1^) at 24 h of exposure. The concentration range in which the *colloidal singularity* occurred is marked in blue. (b) Intracellular ROS formation as DCF fluorescence (% with respect to control levels) and (c) % of *P. subcapitata* cells showing DNA fragmentation, when exposed to representative nZVI concentrations (0, 0.075, 0.5, 2.5 mgL^−1^) at relevant exposure times (0 h, 24 h, 48 h and 72 h). (d) Representative *FS-SS* distribution plots of *P. subcapitata* cells exposed to 0, 0.075, 0.5, 2.5 mgL^−1^ nZVI at relevant exposure times (0 h, 24 h, 48 h and 72 h). Red dots depict cells undergoing cell cycle progression, and blue dots depict cells showing DNA fragmentation (e) Representative cell cycle frequency histograms of *P. subcapitata* cells exposed to 0, 0.075, 0.5, 2.5 mgL^−1^ nZVI at relevant exposure times (0 h, 24 h, 48 h and 72 h). F: cell population containing less DNA than G0/G1 phase, which denoted a cell population showing DNA fragmentation. Black chart: cell population undergoing an abnormal cell cycle (poorly defined S and G2/M phases). Statistically significant differences (*p*<0.05) are marked by asterisks.

EHS assessment of nZVI suspensions was evaluated using an algal growth inhibition assay based on *Pseudokirchneriella subcapitata* (*P. subcapitata*) [18]. The assay consisted of monitoring the algal growth rates based on three different biomass end-points. Besides growth inhibition, a study of alterations in intracellular levels of reactive oxygen species (ROS) and cell cycle was also performed. All biomass related end-points tested correlated (R>0.9, *p*<0.05) (**Figure 3.b, c**) and showed non-linear dose-effect relationships (**Figure 3.a**) which mimicked the ζ-potential and particle size profiles showed in **Figure 2.a, b**. The biological response of the test model organism showed two statistically significant (*p*>0.05) regions of toxic response, bracketing a non-toxic region. The first toxic dose region occurred within nZVI concentrations of 0.05 to 0.075 mgL^−1^, resulting in almost 40% maximum growth inhibition. The second window of concentrations resulting in adverse biological effects ranged from 1.0 to 2.5 mgL^−1^, resulting in almost 60% maximum growth inhibition (**Figure 3.a**). The observed toxic response was consistently accompanied by a statistically significant (*p*>0.05) increase in intracellular ROS production, and cell cycle alterations (**Figure 4.a–e**). ROS production reached nearly 180% and 250% induction with respect to control levels for 0.075 mgL^−1^ and 2.5 mgL^−1^ nZVI, respectively (**Figure 4.a**). Cell cycle alterations consisted of two levels of abnormalities in cell cycle progression of cell populations. Firstly, nearly 50% of the cell population accumulated increasing intracellular DNA quantities. This means that cells accumulated in poorly defined S and G2/M phases (Black chart in **Figure 4.e**). Secondly, almost 30% of cell population showed DNA fragmentation (Blue spots in **Figure 4.d** and F region in **Figure 4.e**) which is usually linked to apoptosis [22], [23]. ROS induction, cell cycle alterations and DNA fragmentation were clearly evident and statistically significant (*p*<0.05) from early stages of nZVI exposure (24 h) (**Figure 4.e, f**). Interestingly, between the two toxic end dose regions, there was a non-toxic dose region within a narrow range of nZVI concentrations (0.1 to 0.5 mgL^−1^), coinciding with the *colloidal singularity* described earlier (**Figure 2**).

**Figure 5:**
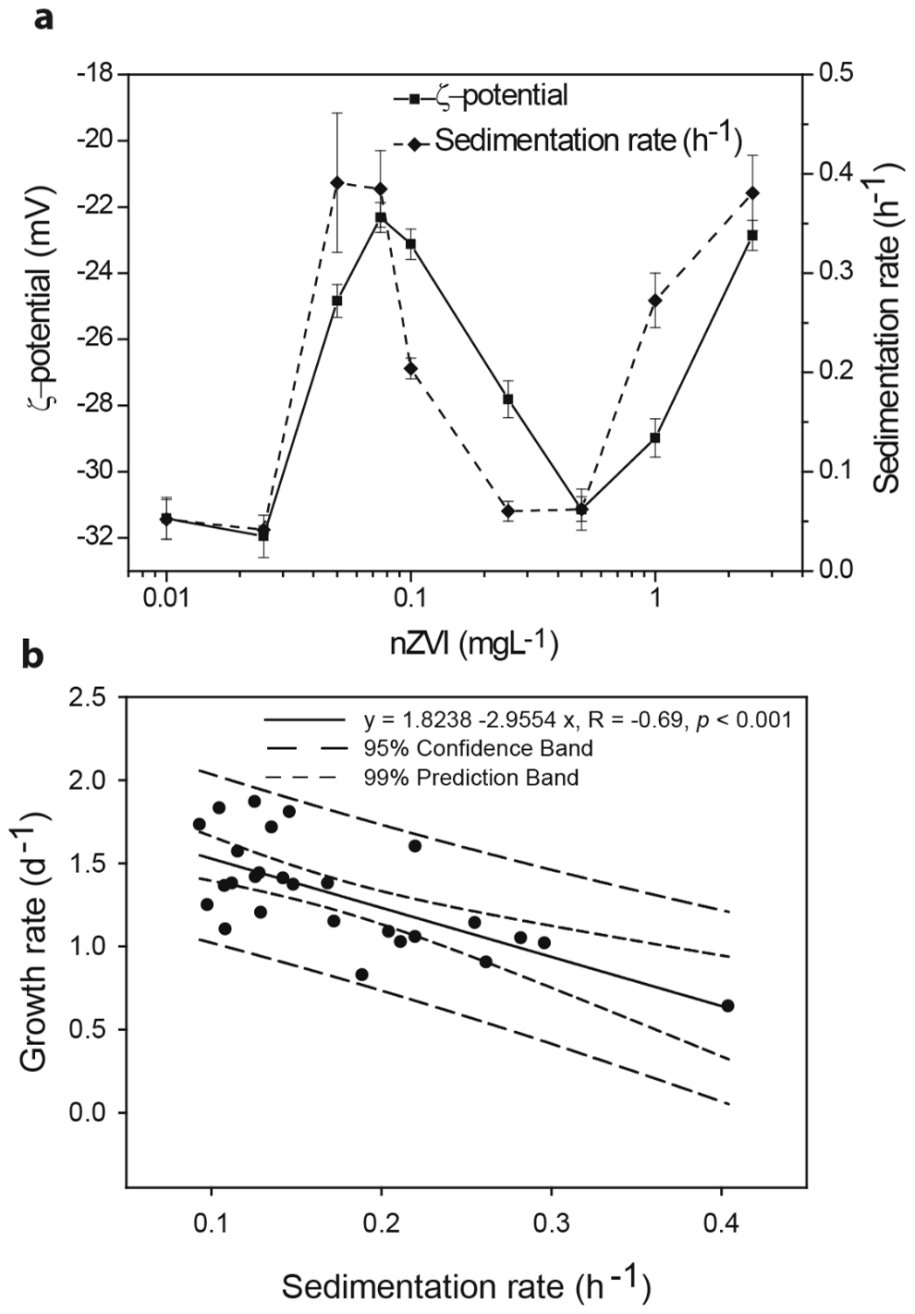
Toxicity of nZVI is mediated by colloidal destabilization and hetero-aggregation between algal cells and nZVI. (a) Sedimentation rates/ζ-potential of test systems exposed to increasing concentrations of nZVI. (b) Correlation of sedimentation rates *vs* algal growth rates.

**Figure 6:**
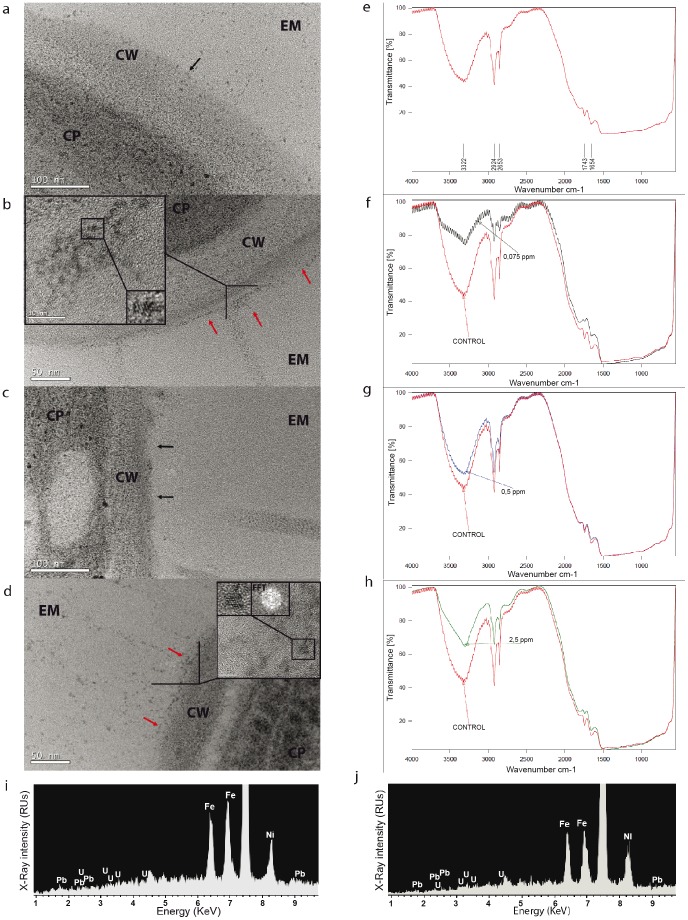
Tracking nZVI physicochemical interactions at the bio-nano interface. Representative TEM images showing the interfacial space delimiting algal cells and the external microenvironment of untreated *P. subcapitata* control cells (a), and *P. subcapitata* cells exposed to 0.075 mgL^−1^ (b), 0.5 mgL^−1^ (c) and 2.5 mgL^−1^ (d) of nZVI during 72 h. EM: external microenvironment, CW: Cell wall, CP: Cytoplasm. Black arrows show the CW-EM interface and red arrows the nZVI attached to the outer region of the algal CW in (b) and (d). Insets in (b) and (d) show CW-EM interfacial regions where surface-bound nZVI accumulations were observed on the CW and where nZVI particles were identified. nZVI particles showed round shapes and near squared lattices. Interplanar spaces (2.8 A° and 3.2 A°) are consistent with those of hematite and iron (III) oxide-hydroxide. Fast Fourier Transform (FFT) pattern of the nanoparticle in inset of (d) revealed a truncated octahedral tridimensional structure [56]. Representative FTIR transmission profile of untreated *P. subcapitata* control cells (e) and *P. subcapitata* cells exposed to 0.075 mgL^−1^ (f), 0.5 mgL^−1^, (g) and 2.5 mgL^−1^ (h) of nZVI during 72 h. Wavenumber (cm^−1^) of absorption peaks vibrations analyzed in the study are marked in (e). (i, j) XEDS spectra of the insets in b and d, respectively. Fe is the main constituent; Ni and Cu intense signals in the XEDS spectra (when present), are artifacts due to the metallic grid supporting the samples, and of the sample holder of the TEM instrument, respectively.

The nZVI-algal suspension can be regarded as a whole colloidal system and its stability properties can be studied as such. Destabilization of colloidal systems is typically accompanied by an increase in sedimentation rates, and a decrease (in absolute value) in ζ- potential which typically account for aggregation and sedimentation of colloidal particles [11], [24], [25]. **Figure 5.a** shows that the algae-nZVI systems exhibited clear signs of colloidal destabilization with the exception of the concentration range in which the colloidal singularity occurred. Furthermore, the decrease in algal growth rates clearly correlated with the sedimentation rates of the test systems (R = −0.689, *p*<0.001) (**Figure 5.b**). This suggests that the toxicity of nZVI is mediated by colloidal destabilization and hetero-aggregation between algal cells and nZVI. To test this hypothesis, events occurring at the bio-interface delimiting algal cells and the external microenvironment were studied by TEM and FTIR analysis (**Figure 6**.). As shown in **Figure 6.b, d**, the presence of electron-dense particles surrounding the external cell wall of the micro algae was identified at 0.075 mgL^−1^ and 2.5 mgL^−1^ of nZVI. However, they were absent in control cells and 0.5 mgL^−1^ nZVI treatment (**Figure 6.a**, **c**, respectively). XEDS analysis assigned Fe as the main constituent of those particles (**Figure 6.i, j**). Furthermore, FTIR analysis (**Figure 6.f and h**) revealed significant changes to the IR signals of algal cell where cell-nZVI attachment occurred (0.075 mgL^−1^ and 2.5 mgL^−1^ nZVI treatments). These changes consisted of a significant reduction of peak absorption at wavenumbers characteristic of asymmetric (2924 cm^−1^) and symmetric (2853 cm^−1^) sp^3^ C-H stretching vibrations, O-H stretching (3222 cm^−1^) and bending (1654 cm^−1^) vibrations in hydroxyl functional groups and C = O stretching vibration (1743 cm^−1^) of carbonyl functional groups [26]. Interestingly, IR signals remained almost unchanged in the 0.5 mgL^−1^ treatment (**Figure 6.g**) compared to control signal (**Figure 6.f**).

### Colloidal destabilization reverts the colloidal singularity and induces nZVI toxicity

**Figure 7:**
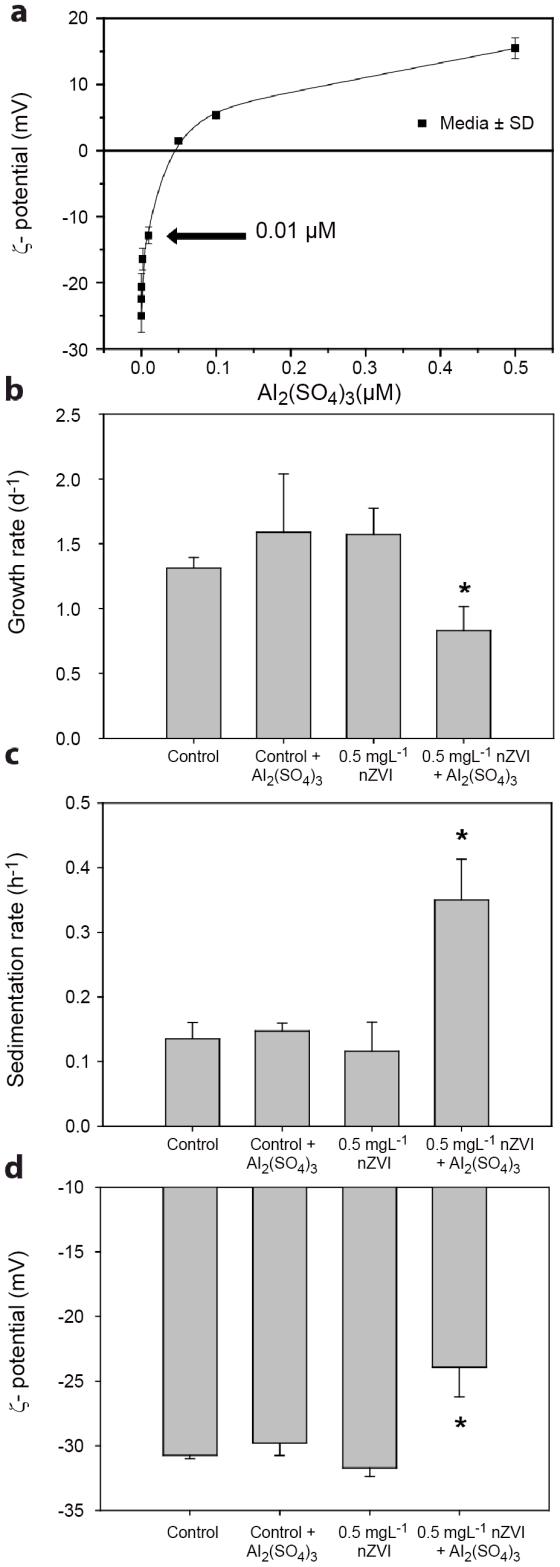
Colloidal destabilization reverts the *colloidal singularity* and induces nZVI toxicity. (a)Titration curve showing an exponential growth profile. Experimental ζ-potential of 0.5 mgL^−1^+nZVI Al_2_(SO_4_)_3_ 0.01 µM is marked by an arrow. (b,c,d) Growth rates, sedimentation rates and ζ-potential (respectively) of colloidal test systems of *P. subcapitata* exposed to 0.5 mgL^−1^ of nZVI in the presence or absence of 0.01 µM Al_2_(SO_4_)_3_. Statistically significant differences (*p*<0.05) are marked by an asterisk.

To further demonstrate the key role played by colloidal stability in the observed lack of toxicity in the colloidal singularity dose range, a destabilization experiment was performed. Destabilization of the *colloidal singularity* was induced by neutralizing the negative surface charge density of the 0.5 mgL^−1^ nZVI suspension by using a classical hydrolyzing metal coagulant [Al_2_(SO_4_)_3_] [11], [24]. Al^3+^, like other polyvalent metal cations, is specifically adsorbed on negatively charge surfaces, thereby reducing the surface charge density [11]. Based on titration experiments with Al_2_(SO_4_)_3_ (**Figure 7.a**), 0.01 µM Al_2_(SO_4_)_3_ was chosen, as it proved to effectively reduce the ζ-potential of the 0.5 mgL^−1^ nZVI suspension from −28 mV to nearly −10 mV, resulting in partial colloidal destabilization. This Al_2_(SO_4_)_3_ had no detrimental effect on the growth of *P. subcapitata* [27] (**Figure 7.b**), nor statistically significant effects on monitored parameters compared to control systems (**Figure 7.c,d**). The addition of Al_2_(SO_4_)_3_ to the test system containing 0.5 mgL^−1^ nZVI caused 2-fold reductions in the growth rates of *P. subcapitata* (*p*>0.05) (**Figure 7.b**). This toxic response was accompanied by a statistically significant (*p*>0.05) increase in sedimentation rates (**Figure 7.c**), and a statistically significant ζ-potential reduction of the nZVI-algae colloidal system (*p*>0.05) (**Figure 7.d**).

## Discussion

When suspensions of nZVI were prepared in OECD culture medium, we observed an ENM speciation phenomenon [3] as a function of dose. We denoted it as *colloidal singularity* since such a non-monotonic speciation phenomenon as a function of dose has never been previously reported. Interestingly, the ζ-Potential/particle size *vs* nZVI dosage diagrams resembled those of an inverted classical flocculation diagram [11], [28]. In the classical flocculation diagram a fixed amount of suspended colloids is exposed to increasing concentrations of a hydrolyzing salt. The multivalent cations of the hydrolyzing salt (commonly Al^3+^ or Fe^2+^) specifically adsorb on the negatively charged surface of the suspended colloids. This results in a characteristic non-linear behavior on the residual turbidity of the suspensions as a function of the resulting colloid/ions ratio. The classical flocculation diagram presents three differential stages: (1) in a first stage, the dose of coagulant is not enough to destabilize the colloids which remained negatively charged and stable. (2) With increasing coagulant concentrations, the adsorbed cations effectively reduce the surface charge of suspended colloids, resulting in colloidal destabilization and removal of colloids. This typically happens in a very narrow coagulant dosage [11], [24]. (3) A further increase in coagulant dose produces re-stabilization of colloidal particles by charge reversal resulting in a stable colloidal system. The success of the coagulation process depends entirely upon the adequate shaking [11], [24]. In our case, two regions of colloidal destabilization bracketing a stable one appeared with increasing nZVI concentration. In contrast with the classical flocculation diagram, we have a fixed ion concentration (from the culture medium) and a variable number of colloidal particles. However, we also generated a variable colloid/ions ratio, which may result in a similar asymmetry in the colloidal stability of the suspensions. Interestingly, in the ζ-potential/particle size *vs* nZVI dose diagrams, we did not find charge reversal causing stabilization of nZVI in the colloidal singularity, but rather an increase in its electrophoretic mobility (ζ-potential). We hypothesized that the high affinity of anions to be adsorbed on nZVI surfaces [17] may promote an increased anion adsorption on the nZVI surface at the specific nZVI/ion ratios established in the *colloidal singularity* dose range This would result in a net increase in negative surface charge and may cause a reduction of its net surface energy, and of its tendency to interact with bio-interfaces.

The occurrence of the colloidal singularity is highly illustrative as a case study for EHS of ENMs. It clearly exemplifies the extreme relevance of ENMs speciation in their ability to interact with living organisms, resulting in differential biological effects [1], [3]. When nZVI appeared stable in its primary size (in the *colloidal singularity*), no detrimental biological effects could be observed. This was true even when its total surface area was higher than that of any aggregated nZVI suspension. This is in apparent disagreement with previous reports [29], [30] and the general consensus: a higher total surface implies a higher exposure, and hence bioactivity [2], [4], [5]. Here, the key point to reconcile the findings is to consider colloidal stability. The concomitant increase in the negative surface charge of nZVI suspensions implies stable colloidal systems. These systems do not cause biological effects due to their lack of tendency to interact with bio-interfaces [8], [25], [31]. This is true regardless of considerations on their surface area or their intrinsic bioactivity. It was further confirmed by the observed correlation between toxicity and hetero-aggregation tendency, and the destabilization experiment with Al_2_(SO_4_)_3_.

Nanoparticle-induced oxidative stress caused by the formation of reactive oxygen species (ROS) has been established as one of the most common paradigms involved in the toxic responses induced by engineered nanoparticles in living organisms [7], [8], [32], [33]. According to this paradigm, when the cellular antioxidant barriers are surpassed, ROS injury can ultimately induce cell death via alterations in cell cycle [33], [34] and the activation of apoptotic signaling pathways [7], [33]. Our results demonstrate that the toxicity of nZVI to *P.subcapitata* is linked to the production of ROS, cell cycle alterations and pre-apoptotic DNA fragmentation; and therefore can be assigned to the oxidative stress toxicity paradigm [5], [7], [33]. Furthermore, toxicity, ROS and cell cycle alterations are mediated by direct attachment of nZVI particles to the outer cell envelopes of the microalgae, which was confirmed independently by TEM-XEDS and FTIR. To our knowledge, this is the first time that the mechanisms of toxicity of nZVI to a microalga have been elucidated. Keller et al. [35] described the toxicity of several commercial bare and polymer-coated nZVI to several marine and freshwater *microalgae*, including *P. subcapitata*. However, the investigation of the underlying toxic mechanism was beyond the scope of their study. ROS mediated toxicity of nZVI is consistent with previous studies with other biological model systems, including bacteria, fish and mammals [8], [16], [36]–[40]. Similarly, toxicity mediated by direct attachment of nZVI to the cell surface is also well documented for bacteria [8], [16], [38], [39], [41]. FTIR spectra found in *P. subcapitata* control cells markedly resemble those of cyclic sugars [42]–[44] such as those found in algal cells walls, mainly cellulose, hemicellulose and pectins. The alterations in those peak vibrations when nZVI presented toxicity may confirm a direct alteration of the main chemical bonds of *P. subcapitata* cell wall. This suggests the presence of active binding sites of nZVI to *P. subcapitata* cell wall. The latter may imply that nZVI is able to induce intracellular specific toxic responses by damaging the structure and changing the physicochemical properties of surface biomolecules [45]. Jiang et al. [45] found that oxide nanoparticles (Al_2_O_3_, TiO_2_ and ZnO) induced chemical and structural changes on bacterial cell envelopes, and that toxicity correlated with the severity of those changes. Similarly, other authors have found direct bond-interaction of oxide nanoparticles with algal and bacterial cell walls mediating toxicity and ROS [46]–[52]. The work by Gong et al. [46] on the toxicity of NiO nanoparticles to the microalgae *C. vulgaris* is especially interesting. They found that toxicity of NiO nanoparticles was mediated by hetero-aggregation. Furthermore, they found a decrease in the NiO signal and the appearance of a Ni° peak in the X-Ray diffraction spectra of NiO nanoparticles attached to the cell walls. This evidenced that a redox reaction was occurring between the NiO surface and the cell walls, resulting in the reduction of Ni (II) to Ni° [46]. The counterpart of the reduction is the oxidation of surface biomolecules of the organism resulting in chemical bonding. Our FTIR results and the fact that nZVI is covered by layers of iron oxy-hydroxides under oxidant conditions (Figure S**1**) [17], led us to think that the ROS production mechanism proposed for nZVI, *i.e.*, a Fenton/Haber–Weiss reactions [37]–[40], would not be the main mechanism of action responsible of ROS induction in *P. subcapitata*. We hypothesize that the spontaneous bio-reduction of some of the surface iron oxy-hydroxides by microalgal surface biomolecules, might be the origin of the attachment of nZVI to *P. subcapitata*. This may result in concomitant ROS induction and cell cycle alterations which is in agreement with the oxidative stress paradigm of redox cycling at the bio-interface [53]. However, this hypothesis requires further investigation.

From our results, it can be inferred that colloidal stability is a crucial regulator of the interaction of negatively charged ENMs with bio-interfaces. In this regard, there is a huge number of published research reporting toxicity of a variety of negatively charged metallic and carbonaceous ENMs on aquatic organisms [8], [10], [51], [52]. A relevant part reported hetero-aggregation [8], [10], [15], [51], [52], despite the known negative surface charge of living cells [8], [9]. This suggests a key role of negative-negative colloidal interactions in the fate and biological effects of ENMs in aquatic systems, which may be especially relevant for *microalgae* and other aquatic microorganisms in the “colloidal” size region. From our results, surface charge seems to play a major role in these negative-negative interactions because an increase (in absolute value) of nearly 10 mV (from −15 mV to −25 mV) seems to be enough to prevent hetero-aggregation. Similarly, a reduction from −25 mV to −14 mV was enough to induce it. However, would all ENMs and organisms have the same intrinsic tendency to generate hetero-aggregation? Logic seems to indicate that probably not. However, the rules governing the interactions between negatively charged colloids and organism interfaces remain unclear [8], and at present, no systematic studies concerning this issue have been reported.

From a basic methodological view-point, our findings have some broader implications for EHS evaluation of ENMs *in-vitro*. Firstly, when performing EHS screening [5], the need to achieve stable ENM suspensions, by stabilizing agents, polymers, etc., may yield false negative bioactivity results [5] if the ENM suspensions under evaluation are excessively stabilized. Secondly, overlooking colloidal stability may have severe implications in basic aspects of EHS characterization of ENMs. For example, characteristic-dependent ENM bioactivity, when comparing different sizes, shapes, aspect ratio, redox states, etc., can be masked due to a heterogeneous degree of colloidal destabilization of the working suspensions, resulting in misleading conclusions.

## Conclusions

The influence of physicochemical state on the bioactivity and toxicity of ENMs is presently under discussion. In the present work, when a linear nZVI dose-range was prepared for EHS characterization, a spontaneous speciation phenomenon occurred which resulted in a qualitatively different physicochemical state of nZVI along the dose gradient. We called it *colloidal singularity*. Basically, nZVI generally appeared in the form of aggregates. However, there was a narrow dose-range (the *colloidal singularity*), between 0.1–0.5 mgL^−1^ nZVI where it appeared highly stable (low particle size and increased ς-potential). nZVI resulted in adverse biological effects when destabilized, but not when stable (the *colloidal singularity*). Furthermore, destabilization of those particular suspensions resulted in toxicity. Considering colloidal stability as a driver of bio-physical interactions allows us to explain our findings and previous apparently contradicting reports. In summary, our work demonstrates the role played by colloidal stability as a fundamental driver of ENM bioactivity, and opens up new perspectives on the relevant factors which may be considered in EHS and bioactivity testing of ENMs.

## Experimental Section

### nZVI and chemicals

All chemical reagents in the study were of reagent grade and used as purchased without further purification or pretreatment. Nano zero-valent iron (nZVI) powder was purchased from Quantum-Sphere (California, USA). The water used throughout the work was Milli-Q water. nZVI stock suspensions (25 mgL^−1^) were prepared in Milli-Q water followed by a 10-minute sonication bath (Ultrason Selecta, Spain) to avoid particle agglomeration or aging. 25 mgL^−1^ of nZVI in Milli-Q water was selected as stock suspension since it had the lowest polydispersity index (PDI) and effective diameter (**Table 1**) and was sufficiently concentrated to be used as stock suspension for further EHS testing. Stock suspensions were always freshly prepared a few minutes prior to any physicochemical or biological experiment in order to ensure homogeneity of stock suspensions and prevent differences in aging and/or aggregation state throughout the different physicochemical and biological studies.

The current use of nZVI particles in the remediation of water resources contaminated with recalcitrant organic pollutants is based on *in situ* injection of large quantities (up to 50 g nZVI/L) into the subsurface waters [40]. Following removal from solution via sedimentation and dispersal with water flow, a nZVI concentration gradient would develop downward from the point of injection and much lower concentrations as one moves away from the point of injection. While it is still unclear what the actual nZVI concentrations are along such a gradient, in this study, we used what could reasonably be concentrations in the low end of such a gradient.

### Physicochemical characterization of nZVI stock suspensions

The Nova 1200 BET (Brunauer –Emmett –Teller) was used to determine the surface area of pristine nZVI. Morphology properties were studied by Transmission Electron Microscopy (TEM) at 200 KeV operating voltage in a JEOL JEM 2100 electron microscope with 0.25 nm point resolution coupled with XEDS (X-Ray Energy Dispersive Spectroscopy). ENMs size measurements, interplanar spacing of crystalline planes and Fast Fourier Transform (FFT) power spectrums were analyzed using Digital Micrograph software, 2.30.542.0 (Gatan Inc.). The size distribution of nZVI was obtained using dynamic light scattering (DLS, Malvern Zetasizer Nano ZS). Measurements were conducted performing 500 rounds measurements and in particle number mode. ς-potential was measured via electrophoretic light scattering combined with phase analysis light scattering in the same instrument. ζ-potential was derived from electrophoretic mobility by applying the Henry equation and the Smoluchowski approximation [11]. Measurements were conducted at ambient temperature (25°C) in Milli-Q water and/or OECD TG 201 assay media without any modification. DLS measurements were performed in particle number mode. The count rates obtained in DLS and ς-potential measurements were higher than 15 kilo counts per second (kcps) for all the measurements included in analysis. The quality of data was also evaluated by visual observation of autocorrelation curves.

### Physicochemical characterization of stable nZVI fractions during the toxicity experiments

The suspension stability of nZVI particles was measured over time (0, 4, 24, 48 and 72 h) by spectrophotometry (Hitachi U-2000 spectrophotometer, Japan). 250 mL of the desired nZVI concentrations were prepared in OECD TG 201 algal growth medium in 500 mL Erlenmayer flasks (chemical composition of OECD TG 201 algal growth medium can be found in **Table S2**.). Samples were maintained under identical experimental conditions as the bioassays. The temporal stability of nZVI particles was evaluated by measuring the residual absorbance at λ = 750 nm. 25 mL of samples were carefully taken from the supernatant of the test flasks to prevent catching sedimented non-colloidal nZVI fractions. Absorbance measurements were performed in 100 mm light path spectrophotometric glass cuvettes (100-OS, Hellma, Germany). Main physicochemical properties of nZVI colloidal fractions were evaluated by measuring ζ-potential and DLS of the supernatant of the test flasks. Total surface area (SSA) was calculated accordingly to the equation 1 proposed by Macé et al. [54] assuming spherical particles:
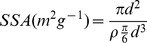
(1)where ρ is the density of the solid particle (6700 Kg m^−3^ accordingly to producer) and *d* is the effective diameter of the particles.

### Growth inhibition experiments using *P. Subcapitata*

The green alga *Pseudokirchneriella subcapitata* (*P. subcapitata*) was purchased from Microbiotests. Inc. (Denmark). *P. subcapitata* was routinely grown in 250 ml flasks at 28°C in light, ca. 65 µmol photons m^2^ s^−1^ on a rotatory shaker at 135 rpm in OECD TG 201 standard algal culture medium [18] (**Table S2**.) (pH 8.2; Conductivity 0.214 µS/cm^2^). Exposure experiments to nZVI suspensions were carried out in 12 mL of OECD TG 201 culture medium in 25 mL glass Erlenmeyer flasks. Before exposure to nZVI, cultures were washed once and re-suspended in fresh culture media to obtain a final optical density (OD_λ_ = _750 nm_) of 0.1. nZVI was added to achieve the desired concentrations, and cultures were exposed for up to 72 h in a rotary shaker at 135 rpm and 28°C under constant illumination. Growth inhibition experiments were performed in triplicate with serial dilutions essentially as described in the standard OECD TG 201 [18]. Three biomass surrogates were measured: OD_λ_ = _750 nm_, total chlorophyll content and *in-vivo* chlorophyll fluorescence. OD_λ_ = _750 nm_ was determined by spectrophotometry in a Hitachi U-2000 spectrophotometer (Japan) by measuring the culture absorbance at λ = 750 nm in 10 mm light path transparent plastic cuvettes. For nZVI nominal concentrations lower than 5 mg/L no interference of nZVI was observed in the determination of OD_λ_ = _750 nm_ of *P. subcapitata* (data not shown). For chlorophyll content determinations, culture aliquots (250 µL) were extracted in methanol at 4°C for 24 h in darkness. The total chlorophyll content of the extract (chlorophyll a+b) was determined by spectrophotometry as described elsewhere [52]. In-vivo fluorescence of chlorophyll was measured daily by transferring 100 µl of quadruplicate samples of cultures to an opaque black 96 well microtitter plate and by measuring fluorescence (485 nm/645 nm excitation/emission) on a Synergy HT multimode microplate reader (BioTek, USA).

### Assessment of ROS generation

Intracellular reactive oxygen species (ROS) produced by *P. subcapitata* was assessed by using the fluorescent probe 2′,7′-dichlorofluorescein diacetate (H_2_DCFDA) (Invitrogen Molecular Probes; Eugene, OR, USA). The intracellular oxidation of H_2_DCFDA generates 2,7-dichlorofluorescein (DCF), a fluorescent compound that serves as an indicator for hydrogen peroxide and other ROS, such as hydroxyl and peroxyl radicals. A 100 mM H_2_DCFDA stock solution was freshly prepared in DMSO under dim light conditions to avoid degradation. Prior to analysis, the green alga was incubated for 30 min at room temperature (23°C) with a final concentration of 100 µM of H_2_DCFDA. As a positive control for ROS formation, 3% H_2_O_2_ (v/v) was used. Fluorescence was monitored on a Synergy HT multimode microplate reader (BioTek,USA) with excitation and emission wavelengths of 488 and 530 nm, respectively. Results were normalized, for differences in cell numbers, by measuring chlorophyll content (as described previously) and expressed as arbitrary fluorescence units (AFU) per µg of chlorophyll a+b.

### Cell cycle and cell population dynamics by flow cytometry

Alterations in cell cycle were assessed by propidium iodide (PI) staining and flow cytometry analysis using a Cytomix FL500 MPL flow cytometer (Beckman Coulter Inc., Fullerton, CA, USA). 1 mL of cell suspensions of the nZVI exposure experiments were collected in 1.5 mL eppendorf tubes and fixed by adding 3% paraformaldehyde (final concentration). Samples were kept in darkness at 4°C until further preparation. For PI staining, cells were gently washed with 1 mL PBS and were incubated for 3 h at 37°C in 1 mL of 0.05% Triton X-100, 0.24 mg/mL RNAase A (all from Sigma-Aldrich) in PBS buffer for membrane permeabilization. Cells were stained in 250 mL of staining buffer consisting of 0.05% Triton X-100, 0.24 mg/mL RNase A and 40 µg/mL PI and were incubated for 24 h in the dark at 4°C. Nuclear DNA content was then analysed using a Cytomics FC500 MPL flow cytometer with the MXP software (Beckman Coulter Inc., Fullerton, CA, USA) equipped with an argon-ion excitation wavelength (488 nm). The flow rate was set at 1 µL s^−1^ and at least 10,000 events (algal cells) were recorded. Light scattered by cells was collected at two angles: *FS* and *SS*, which were used in combination to distinguish between different cell populations. Non-algal particles were excluded from the analysis by setting an acquisition threshold value 1 for the FS parameter. Chlorophyll red autofluorescence was collected with a 675 nm long band pass filter (FL4), PI fluorescence was collected with a 575 nm long band pass filter (FL2), and the signal was adjusted to exclude the residual fluorescence signal coming from the chlorophyll red autofluorescence (FL4) (data not shown). Data acquisition was performed using MXP-2.2 software, and the analyses were performed using CXP-2.2 analysis software. Fluorescence was analysed in Log mode (chlorophyll fluorescence) and linear mode (PI fluorescence).

### nZVI- algal interaction analyses by TEM microscopy and FTIR

For transmission electron microscopy (TEM) analysis, algal cell suspensions exposed to nZVI were collected by centrifugation using a swinging bucket rotor at low relative centrifugal forces (RCFs = 1500 g) during 3 min in order to reduce the chance of artifacts [55], washed three times in phosphate buffer (0.1 M Na-phosphate, pH 7.2) to clean samples and remove unattached nZVI particles. After the final centrifugation step nearly 1 mm of cell pellet was formed. Supernatant was removed and 50 µl of 2% bacteriological ultrapure agar (CONDA, Spain) prepared in phosphate buffer was added to obtain 1 to 2 mm agar blocks. Cells were fixed in 3.1% freshly prepared glutaraldehyde in phosphate buffer (0.1 M Na-phosphate, pH 7.2) for 3 h at 4°C. Postfixation was in osmium tetroxide 1% prepared in phosphate buffer (0.1 M Na-phosphate, pH 7.2) for 2 h at 4°C. The samples were then gradually dehydrated in ethanol during 24 h and embedded in Durcupan resin (epoxy) (Fluka) sectioned (100 nm ultrathin films) in a Leica Reichert Ultracut S ultramicrotome and stained with uranyl acetate 2% in water. Samples were observed on Formvar/carbon Cu grids 300 mesh, at 200 KeV operating voltage in a JEOL JEM 2100 electron microscope with 0.25 nm point resolution coupled with XEDS (X-Ray Energy Dispersive Spectroscopy). All reagents used for TEM preparations were EM grade. Sample preparation for TEM microscopy involves centrifugation and washing of the cells, which may alter nZVI distribution on the algal cells [55]. Therefore, nZVI-cell interactions were further confirmed by IR spectroscopy. For Fourier Transformed Infrared (FTIR) analyses, 1 mL of exposed cells was centrifuged (5500 rpm, 5 min) and supernatant was removed. 5 µL of pelletized cells were transferred to 1 mm^2^ glass cover-slips and were dried for 2 h at 25°C. Infrared spectra of the algal cell before and after nZVI contact experiments were obtained using a Bruker model IFs 66VFourier Transform Infrared (FTIR) spectrometer in transmission mode. This analysis allowed us to track changes in the chemical bonds and to elucidate the chemical groups involved in nZVI sorption processes.

### Colloidal stability of nZVI-*algal* suspensions

Colloidal stability of nZVI-*algal* suspensions was evaluated by measuring ζ-potential, DLS and sedimentation rates of the colloidal system integrated by the algal cells and nZVI. For the determination of the sedimentation rates, a sedimentation system consisting of 15 mL falcon tubes was used. *Algae* exposed to the different nZVI concentrations were collected and OD_λ_ = _750 nm_ were determined by spectrophotometry (Hitachi U-2000 spectrophotometer, Japan). All colloidal systems were leveled to a homogeneous OD_λ_ = _750 nm_ of 0.350 with a final volume of 5 mL, therefore sedimentation rates were neither influenced by differences in initial optical densities nor test symmetry (mainly water column height). Residual turbidity (OD_λ_ = _750 nm_) of the test systems was measured spectrophotometrically as previously described by taking 250 µl of the near meniscus region of the test tubes at specific time intervals (0, 90, 180 min) and measuring the OD_λ_ = _750 nm_. Sedimentation rates were derived from the differences in residual turbidity of the test systems as a function of time according to equation 2.
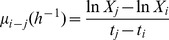
(2)where *μ_i-j_* is the average specific sedimentation rate (h^−1^) from time i to j, X_j_ and X_i_ are the residual optical density at λ = 750 nm (OD_λ_ = _750 nm_) at times i to j; and t_j_ and t_i_ are time (h). ζ-potential of cells exposed to nZVI samples were measured at the end of the exposure experiments. For that, 2 mL of exposed cells were collected and transferred to 2.5 mL eppendorf tubes and ζ-potential was measured directly via electrophoretic light scattering combined with phase analysis light scattering in a Malvern Zetasizer Nano ZS as describe above.

### Colloidal destabilization of nZVI suspensions with Al_2_(SO_4_)_3_

nZVI Destabilization experiments were performed in 15 mL Erlenmeyer flask containing 12 mL OECD TG 201 culture medium. Algal cells were exposed to 0.5 mgL^−1^ of freshly prepared nZVI as described in the toxicity exposure experiments. 0.01 µM of Al_2_(SO_4_)_3_×18 H_2_O (final concentration) was added to the test flask at the beginning of the experiments in order to induce colloidal destabilization and heteroaggregation of the nZVI*-algal* system. Growth inhibition, sedimentation rates and ζ-potential of the colloidal systems were measured as described above. The concentration of Al_2_(SO_4_)_3_ used for the colloidal destabilization experiments was selected based on the results of a titration experiment of 0.5 mgL^−1^ of nZVI suspended in OECD TG 201 algal growth medium with increasing concentrations of Al_2_(SO_4_)_3_ (**Figure 7.a**). 0.01 µM of Al_2_(SO_4_)_3_ was selected due to its ability to partially neutralize the negative surface charges of nZVI (0.5 mgL^−1^) without causing total neutralization or charge reversal.

### Replication and Statistical Analysis

All the experimental procedures were performed at least in triplicate. The mean and standard deviations (SD) were calculated for each parameter. Statistical analyses were evaluated by R statistical program. A one-way ANOVA coupled with Student–Newman–Keuls *post-hoc* test was performed for comparing means of the toxicity effect and physicochemical characteristic among different nZVI concentrations. Statistically significant differences were considered to exist when *p*<0.05.

## Supporting Information

Figure S1 **X-Ray diffraction (XRD) of pristine nZVI powder.** X-ray diffraction analysis of pristine nZVI powder showed peaks of Fe° (Fe) and iron oxide formation of magnetite/maghemite (Fe_3_O_4_/γ-FeOOH) (M) respectively.
(DOCX)

Table S1 **Size of nZVI primary particles and aggregates by TEM microscopy.**
(DOCX)

Table S2 **Chemical composition of OECD TG 201 standard algal culture medium.**
(DOCX)
