# Supplementary figures and images for: A Colloidal Singularity Reveals the Crucial Role of Colloidal Stability for Nanomaterials In-Vitro Toxicity Testing: nZVI-Microalgae Colloidal System as a Case Study
Source: PLoS One. 2014 Oct 23;9(10):e109645. doi: 10.1371/journal.pone.0109645 (PMC4207682; doi:10.1371/journal.pone.0109645)

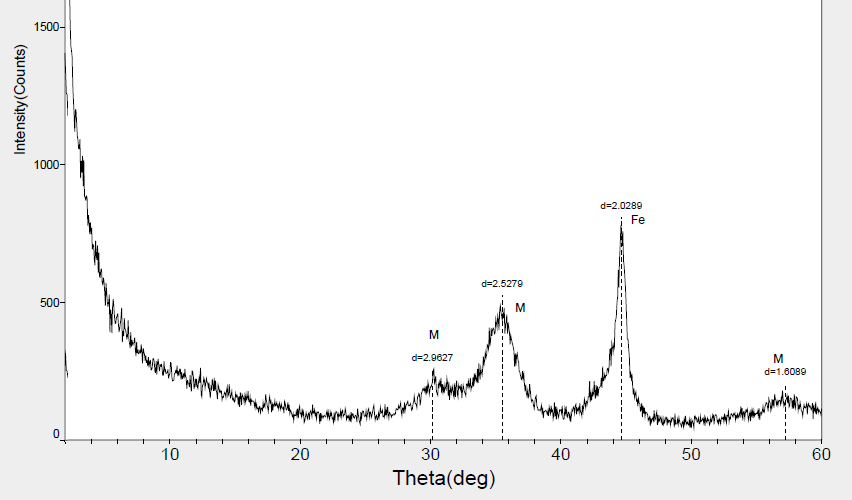

Supplement: Figure S1 — X-Ray diffraction (XRD) of pristine nZVI powder. X-ray diffraction analysis of pristine nZVI powder showed peaks of Fe° (Fe) and iron oxide formation of magnetite/maghemite (Fe3O4/γ-FeOOH) (M) respectively. (DOCX) [file pone.0109645.s001.docx]
