# Supplementary material for: A Colloidal Singularity Reveals the Crucial Role of Colloidal Stability for Nanomaterials In-Vitro Toxicity Testing: nZVI-Microalgae Colloidal System as a Case Study
Source: PLoS One. 2014 Oct 23;9(10):e109645. doi: 10.1371/journal.pone.0109645 (PMC4207682; doi:10.1371/journal.pone.0109645)
Supplement: Table S1 — Size of nZVI primary particles and aggregates by TEM microscopy. (DOCX) [file pone.0109645.s002.docx]

| Particle | Size (nm) | sd |
| --- | --- | --- |
| Small particle | 4.0 | 1.4 |
| Big particle | 12.7 | 1.7 |
| Primary aggregates | 37.5 | 9.7 |

Primary particle size was evaluated by measuring particle size on representative TEM images of 5 mg/L nZVI suspensions in miliQ water. Size measurements were performed using image J software on at least 5 different particles of each group (small particle, big particle and primary aggregates).
