# Supplementary material for: A Colloidal Singularity Reveals the Crucial Role of Colloidal Stability for Nanomaterials In-Vitro Toxicity Testing: nZVI-Microalgae Colloidal System as a Case Study
Source: PLoS One. 2014 Oct 23;9(10):e109645. doi: 10.1371/journal.pone.0109645 (PMC4207682; doi:10.1371/journal.pone.0109645)
Supplement: Table S2 — Chemical composition of OECD TG 201 standard algal culture medium. (DOCX) [file pone.0109645.s003.docx]

| Components | Stock conc. | Final conc. | Final conc. |
| --- | --- | --- | --- |
|  | (gL^-1^) | (mgL^-1^) | (µM) |
| **Solution^1^ 1** |  |  |  |
| NH4Cl | 1.5 | 15 | 280.42 |
| MgCl_2_×6 H_2_O | 1.2 | 12 | 59.02 |
| CaCl_2_×2 H_2_O | 1.8 | 18 | 122.44 |
| MgSO_4_×7H_2_O | 1.5 | 15 | 60.85 |
| KH_2_PO_4_ | 0.16 | 1.6 | 11.75 |
| **Solution^1^ 2** |  |  |  |
| FeCl_3_×6 H_2_O | 0.064 | 0.064 | 0.23 |
| Na_2_EDTA×2 H_2_O | 0.1 | 0.1 | 0.19 |
| **Solution^1^ 3** |  |  |  |
| H_3_BO_3_ | 0.185 | 0.37 | 5.98 |
| MnCl_2_×4 H_2_O | 0.415 | 0.415 | 2.09 |
| ZnCl_2_ | 0.003 | 0.003 | 0.022 |
| CoCl_2_×6 H_2_O | 0.0015 | 0.0015 | 0.0063 |
| CuCl_2_×2 H_2_O | 0.00001 | 0.00001 | 5.8658E-05 |
| Na_2_MoO_4_×2 H_2_O | 0.007 | 0.007 | 0.028 |
| **Solution^1^ 4** |  |  |  |
| NaHCO3 | 50 | 50 | 595.23 |

^1^: Solution: it makes reference to the stock solutions containing each group of macro/micro elements in order to avoid interactions or precipitations during storage.
